# Supplementary material for: The Expression and Activation of the NF-κB Pathway Correlate with Methotrexate Resistance and Cell Proliferation in Acute Lymphoblastic Leukemia
Source: Genes (Basel). 2023 Sep 27;14(10):1880. doi: 10.3390/genes14101880 (PMC10606671; doi:10.3390/genes14101880)
Supplement: Supplementary file 1 [file genes-14-01880-s001.zip › Supplementary_Figures_092623.pdf]

a

CANCER HALLMARK	SIZE	ES	NES	NOM p-val	FDR q-val	FWER p-val
INFLAMMATORY RESPONSE	192	0.7	1.96	0	0	0
ALLOGRAFT REJECTION	199	0.7	1.93	0	0	0
COMPLEMENT	188	0.7	1.84	0	0.001	0.002
TNFA SIGNALING VIA NFKB	198	0.7	1.78	0	0.002	0.006
IL6 JAK STAT3 SIGNALING	87	0.6	1.72	0.002	0.007	0.023
IL2 STAT5 SIGNALING	176	0.6	1.68	0	0.015	0.056
INTERFERON GAMMA RESPONSE	182	0.7	1.66	0.006	0.016	0.066
KRAS SIGNALING UP	195	0.5	1.61	0.004	0.028	0.121
PROTEIN SECRETION	96	0.5	1.59	0.014	0.034	0.152
APOPTOSIS	158	0.5	1.56	0.004	0.048	0.206
APICAL SURFACE	34	0.5	1.52	0.018	0.07	0.277
UV RESPONSE UP	158	0.5	1.5	0.016	0.079	0.323
P53 PATHWAY	197	0.5	1.49	0.012	0.079	0.344
INTERFERON ALPHA RESPONSE	83	0.8	1.49	0.057	0.075	0.35
BILE ACID METABOLISM	101	0.4	1.48	0.036	0.074	0.37
CHOLESTEROL HOMEOSTASIS	62	0.5	1.48	0.006	0.074	0.382
XENOBIOTIC METABOLISM	189	0.4	1.46	0.031	0.086	0.414
COAGULATION	137	0.4	1.46	0.041	0.082	0.417
ANGIOGENESIS	36	0.6	1.45	0.051	0.08	0.425
APICAL JUNCTION	183	0.4	1.45	0.022	0.081	0.444

b

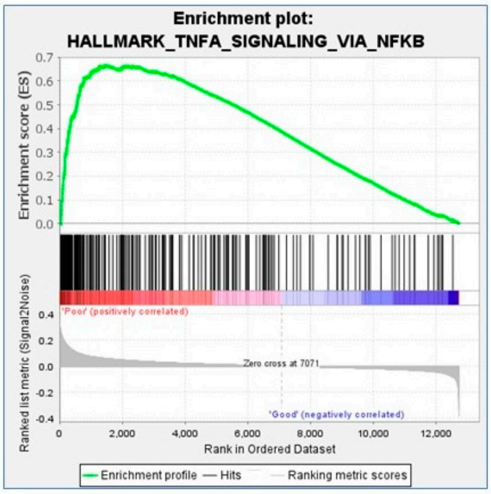

c **d** **e**

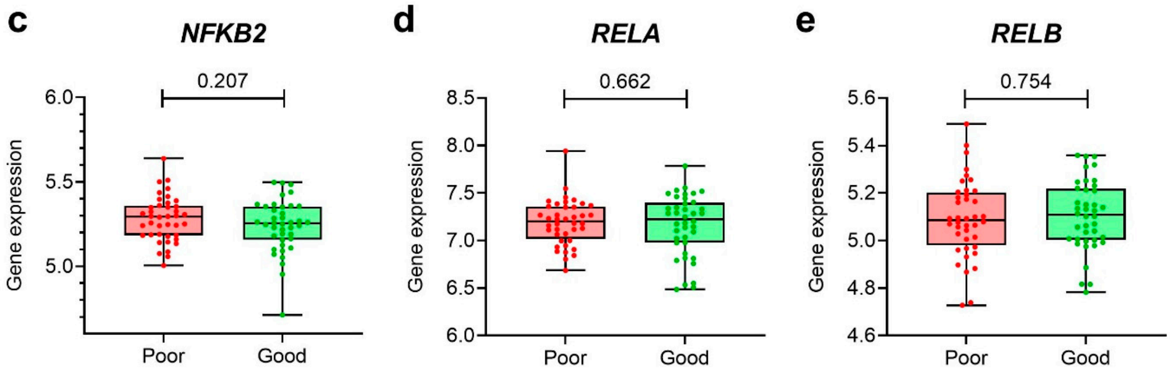

Figure S1. Gene set enrichment analysis (GSEA) on pediatric LLA patients (40 *poor* x 40 *good* responders to MTX). **a**, Cancer Hallmarks most enriched in *poor* responders compared to *good* responders; *TNF-α signaling via NF-κB* is highlighted. **b**, Enrichment plot of the pathway. (**c** – **e**) Expression of the respective NF-κB genes in *poor* and *good* responders (*p*-values for unpaired t-test). *P* values for unpaired t-test. Analysis made on data from Sorich et al. [25] Abbreviations: ALL, acute lymphoblastic leukemia; ES, enrichment score; FDR q-val, false discovery rate *q*-value; FWER p-val, familywise error-rate *P* value; GSEA, gene set enrichment analysis; MTX, methotrexate; NES, normalized enrichment score; Size, number of genes that compose the Hallmark; NOM p-val, nominal *P* value; WBCs, white blood cells.

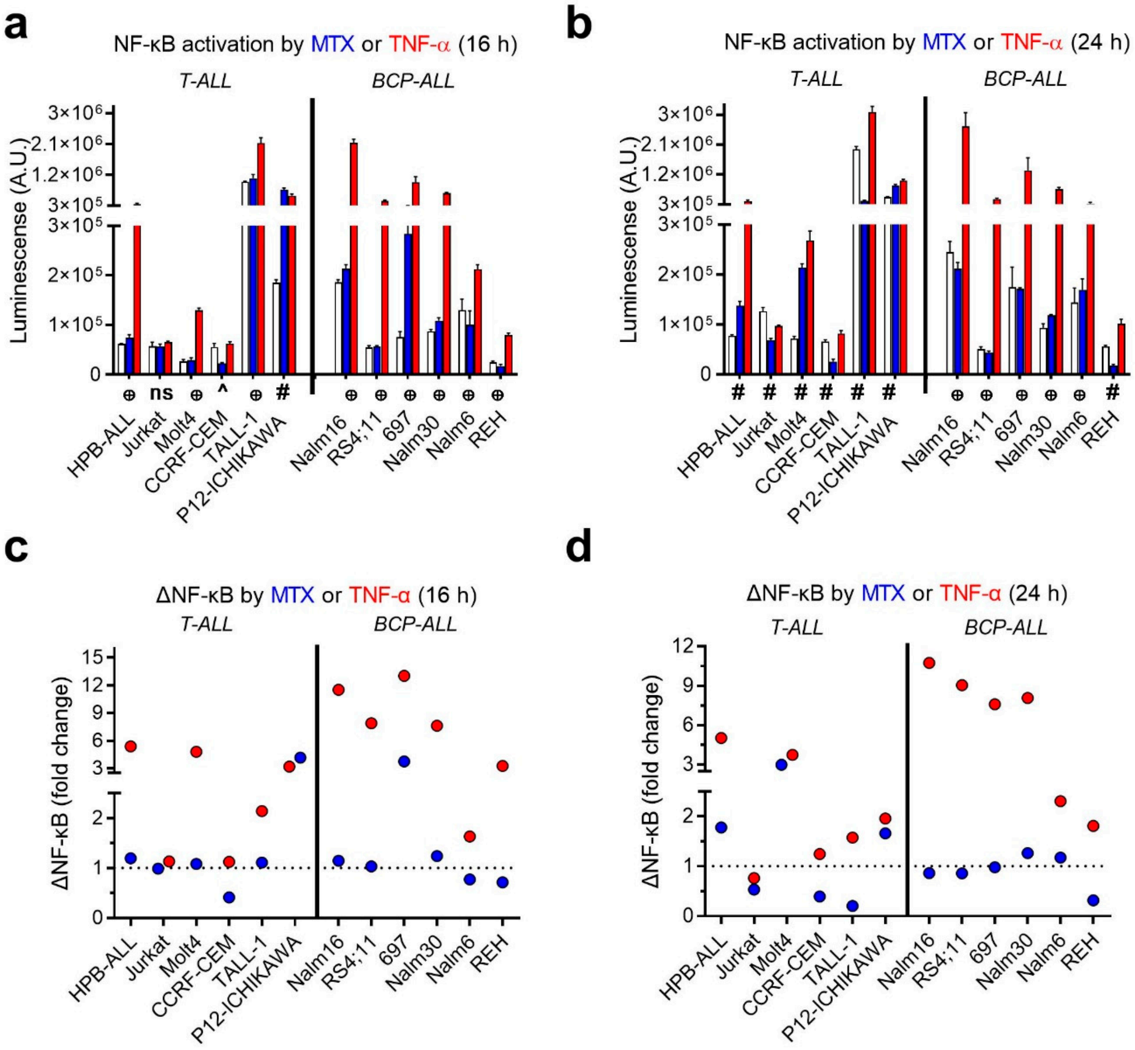

Figure S2. NF-κB activity induced by MTX or TNF-α in transduced ALL cell lines at 16 and 24 hours. NF-κB signal in cell lines treated with MTX (100 nmol/l, in blue), TNF-α (100 ng/ml, in red) or vehicle (Ctrl, white bars) for (**a**) 16 hours or (**b**) 24 hours. One-way analysis of variance followed by Tukey's post-test was performed for each cell line. Symbols: ns: non-significant difference across conditions; ^: MTX differed from both TNF-α and Ctrl, which did not differ between each other; ⊕: TNF-α differed from Ctrl and MTX, which did not differ between each other; #: all three groups differed from one another. **c** and **d**, Ratio between averages in **a** and **b**, respectively, depicting fold change of NF-κB modulation by MTX (blue dots) or TNF-α (red dots) in relation to control (dashed line, normalized per cell line). Abbreviations: ALL, acute lymphoblastic leukemia; A.U., arbitrary units; BCP-ALL, B-cell precursor acute lymphoblastic leukemia; MTX, methotrexate; NF-κB, nuclear factor kappa B; T-ALL, T-cell acute lymphoblastic leukemia; TNF-α, tumor necrosis factor alpha.

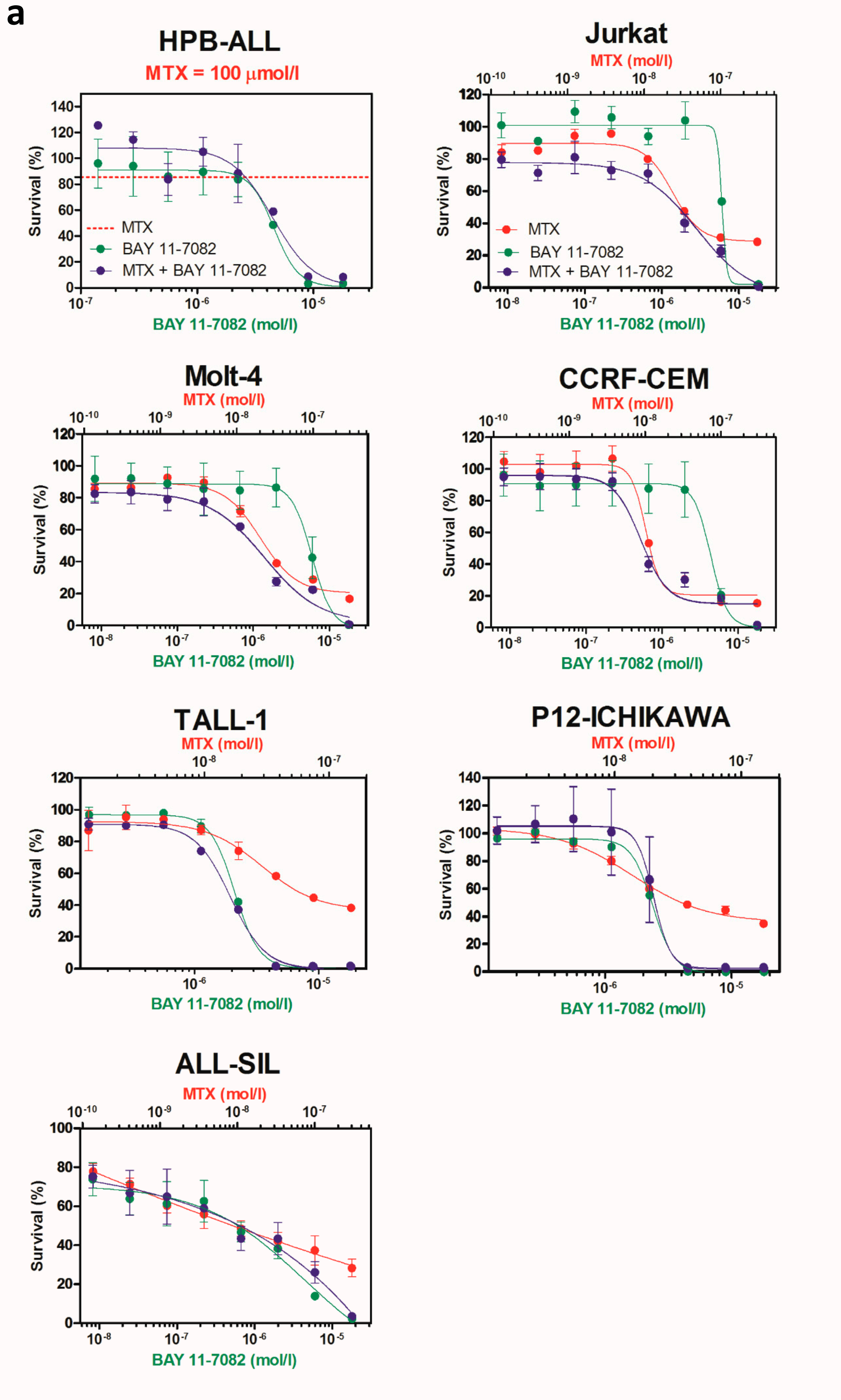
a
HPB-ALL
MTX = 100 µmol/l
Survival (%)
MTX
BAY 11-7082
MTX + BAY 11-7082
BAY 11-7082 (mol/l)
Jurkat
MTX (mol/l)
Survival (%)
MTX
BAY 11-7082
MTX + BAY 11-7082
BAY 11-7082 (mol/l)
Molt-4
MTX (mol/l)
Survival (%)
BAY 11-7082 (mol/l)
CCRF-CEM
MTX (mol/l)
Survival (%)
BAY 11-7082 (mol/l)
TALL-1
MTX (mol/l)
Survival (%)
BAY 11-7082 (mol/l)
P12-ICHIKAWA
MTX (mol/l)
Survival (%)
BAY 11-7082 (mol/l)
ALL-SIL
MTX (mol/l)
Survival (%)
BAY 11-7082 (mol/l)

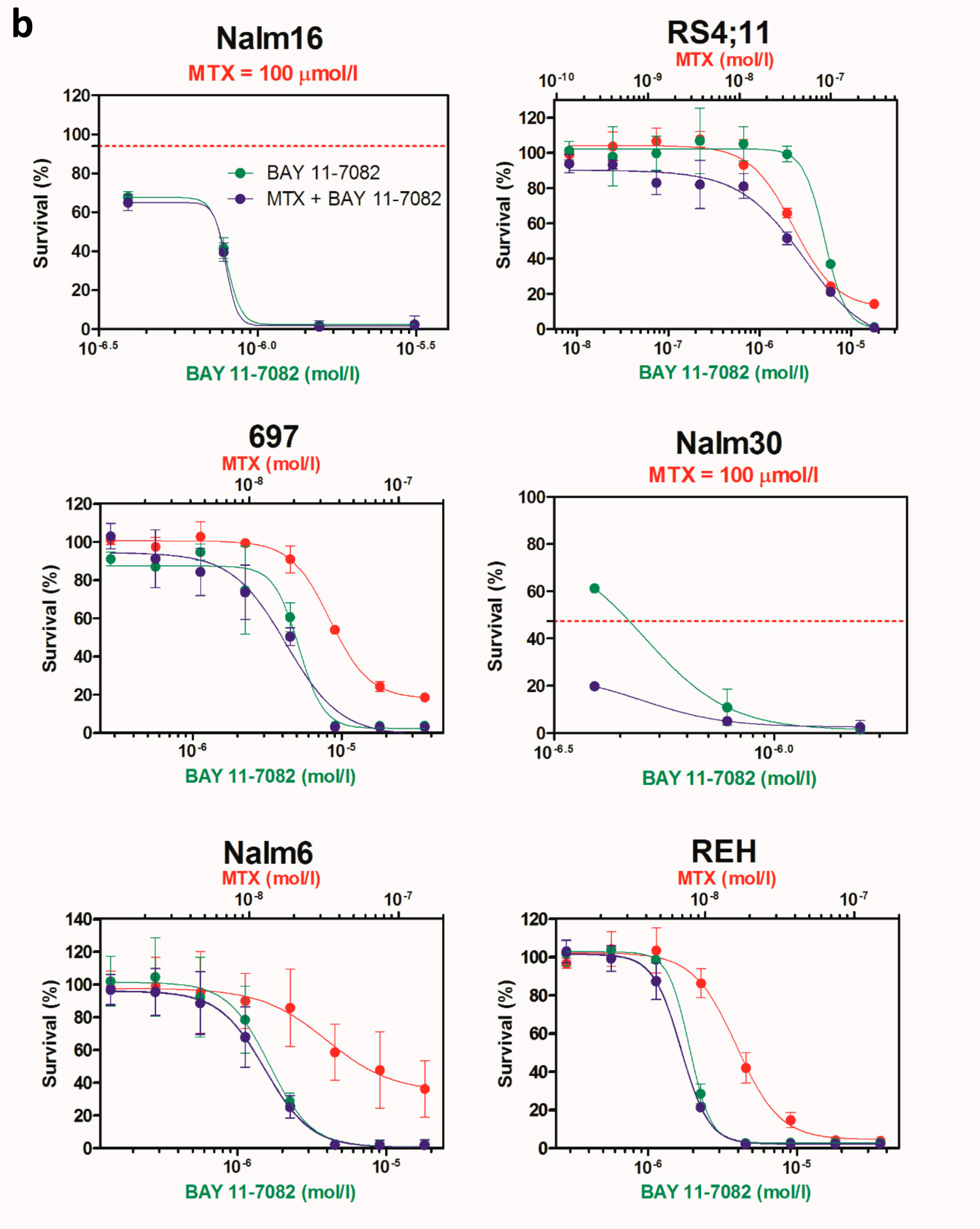
b
Nalm16
MTX = 100 μmol/l
Survival (%)
BAY 11-7082
MTX + BAY 11-7082
BAY 11-7082 (mol/l)
RS4;11
MTX (mol/l)
Survival (%)
BAY 11-7082 (mol/l)
697
MTX (mol/l)
Survival (%)
BAY 11-7082 (mol/l)
Nalm30
MTX = 100 μmol/l
Survival (%)
BAY 11-7082 (mol/l)
Nalm6
MTX (mol/l)
Survival (%)
BAY 11-7082 (mol/l)
REH
MTX (mol/l)
Survival (%)
BAY 11-7082 (mol/l)

c

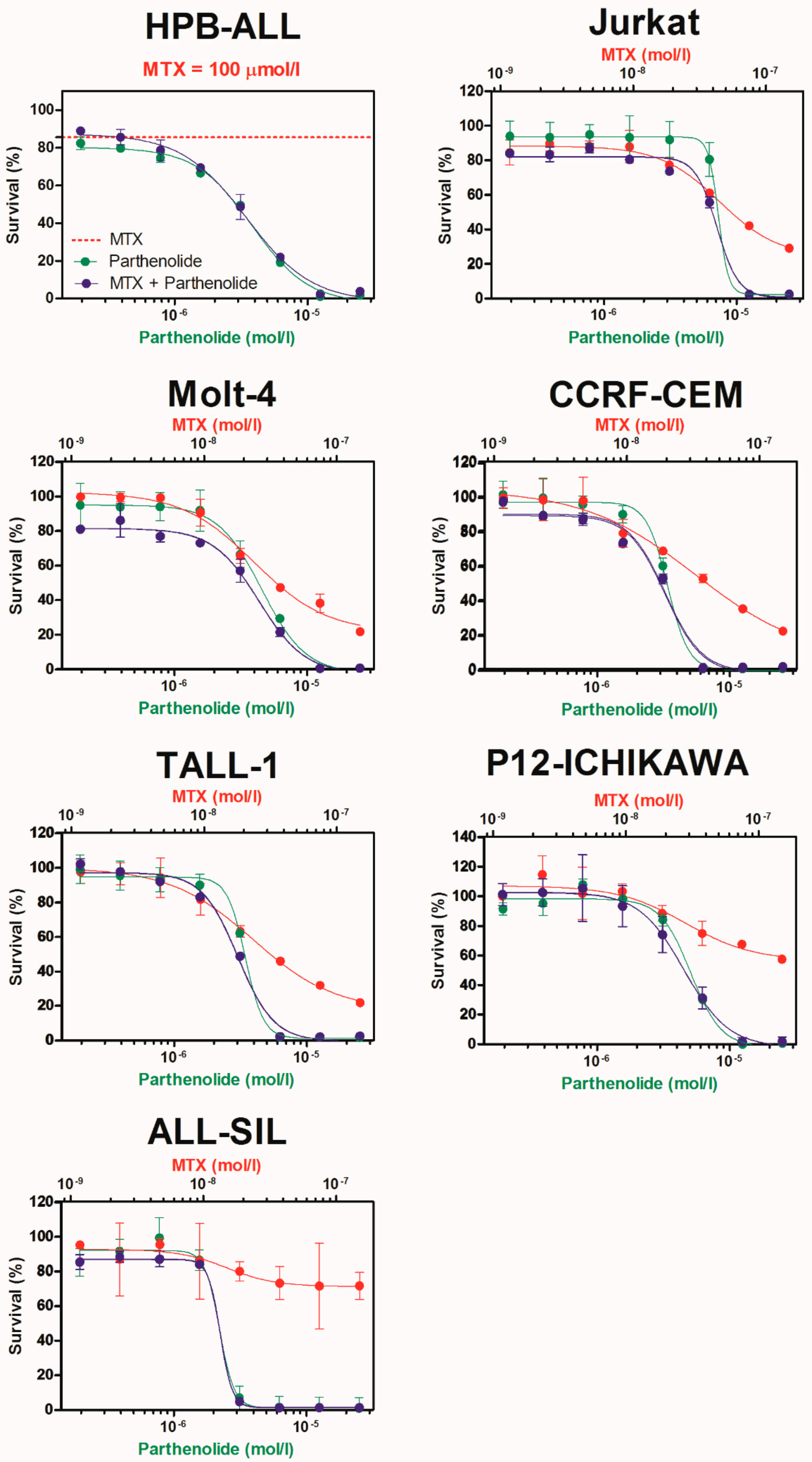

HPB-ALL
MTX = 100 µmol/l
Survival (%)
MTX
Parthenolide
MTX + Parthenolide
Parthenolide (mol/l)
Jurkat
MTX (mol/l)
Molt-4
CCRF-CEM
TALL-1
P12-ICHIKAWA
ALL-SIL

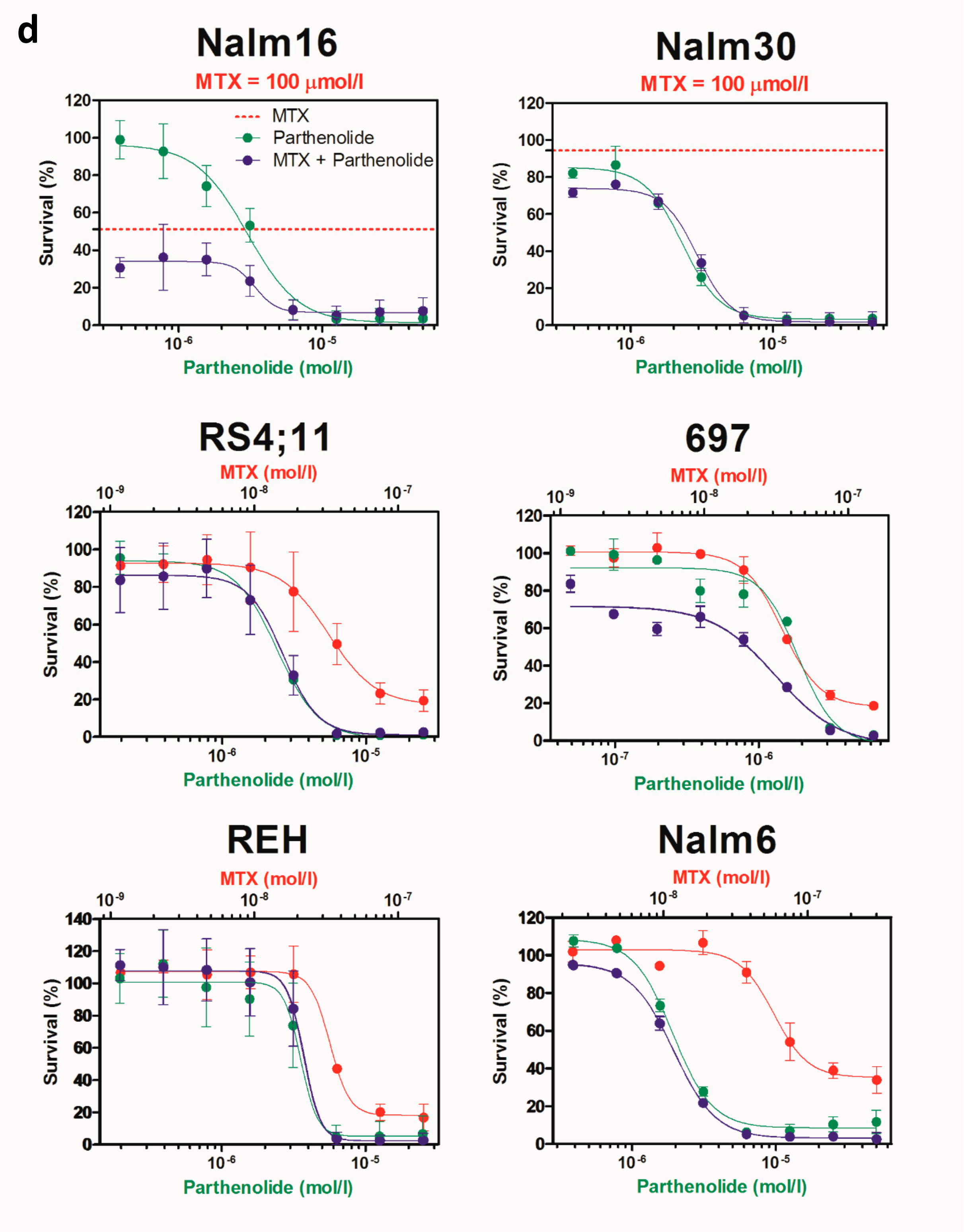

Figure S3. Survival curves to MTX and NF-κB inhibitors (48 hours). **a,** T-ALL cell lines treated with BAY 11 70-82. **b**, BCP-ALL cell lines treated with BAY 11 70-82. **c**, T-ALL cell lines treated with parthenolide. **d**, BCP-ALL cell lines treated with parthenolide. HPB-ALL, Nalm16 and Nalm30 received a constant dose of MTX (100 µmol/l), either alone or combined with increasing doses of the NF-κB inhibitor. Survival is expressed in relation to control (vehicle). Abbreviations: ALL, acute lymphoblastic leukemia; B-cell precursor acute lymphoblastic leukemia; MTX, methotrexate; T-ALL, T-cell acute lymphoblastic leukemia.

a

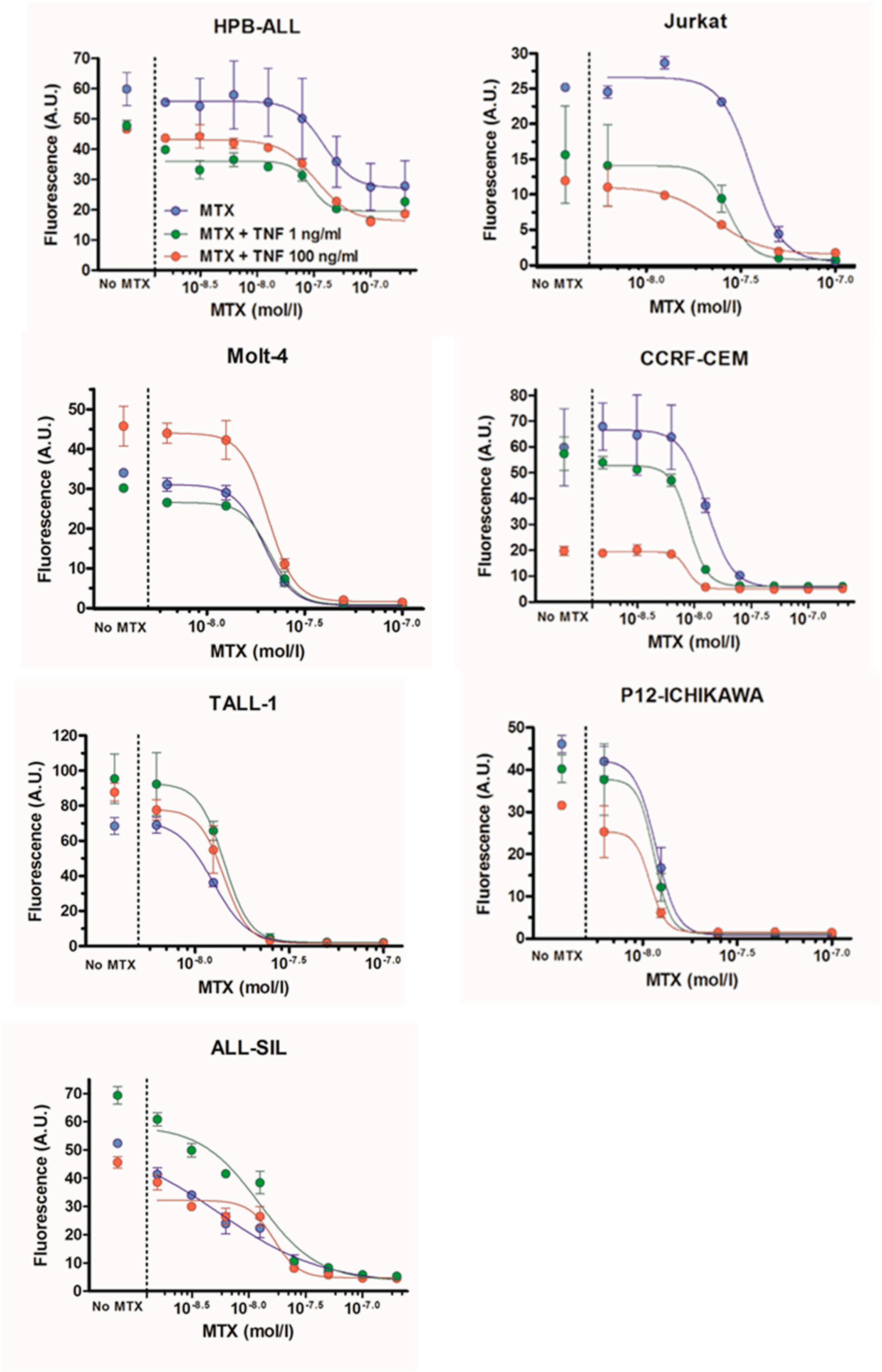
HPB-ALL
Fluorescence (A.U.)
70
60
50
40
30
20
10
0
MTX
MTX + TNF 1 ng/ml
MTX + TNF 100 ng/ml
No MTX
$10^{-8.5}$
$10^{-8.0}$
$10^{-7.5}$
$10^{-7.0}$
MTX (mol/l)
Jurkat
Fluorescence (A.U.)
30
25
20
15
10
5
0
No MTX
$10^{-8.0}$
$10^{-7.5}$
$10^{-7.0}$
MTX (mol/l)
Molt-4
Fluorescence (A.U.)
50
40
30
20
10
0
No MTX
$10^{-8.0}$
$10^{-7.5}$
$10^{-7.0}$
MTX (mol/l)
CCRF-CEM
Fluorescence (A.U.)
80
70
60
50
40
30
20
10
0
No MTX
$10^{-8.5}$
$10^{-8.0}$
$10^{-7.5}$
$10^{-7.0}$
MTX (mol/l)
TALL-1
Fluorescence (A.U.)
120
100
80
60
40
20
0
No MTX
$10^{-8.0}$
$10^{-7.5}$
$10^{-7.0}$
MTX (mol/l)
P12-ICHIKAWA
Fluorescence (A.U.)
50
40
30
20
10
0
No MTX
$10^{-8.0}$
$10^{-7.5}$
$10^{-7.0}$
MTX (mol/l)
ALL-SIL
Fluorescence (A.U.)
70
60
50
40
30
20
10
0
No MTX
$10^{-8.5}$
$10^{-8.0}$
$10^{-7.5}$
$10^{-7.0}$
MTX (mol/l)

b

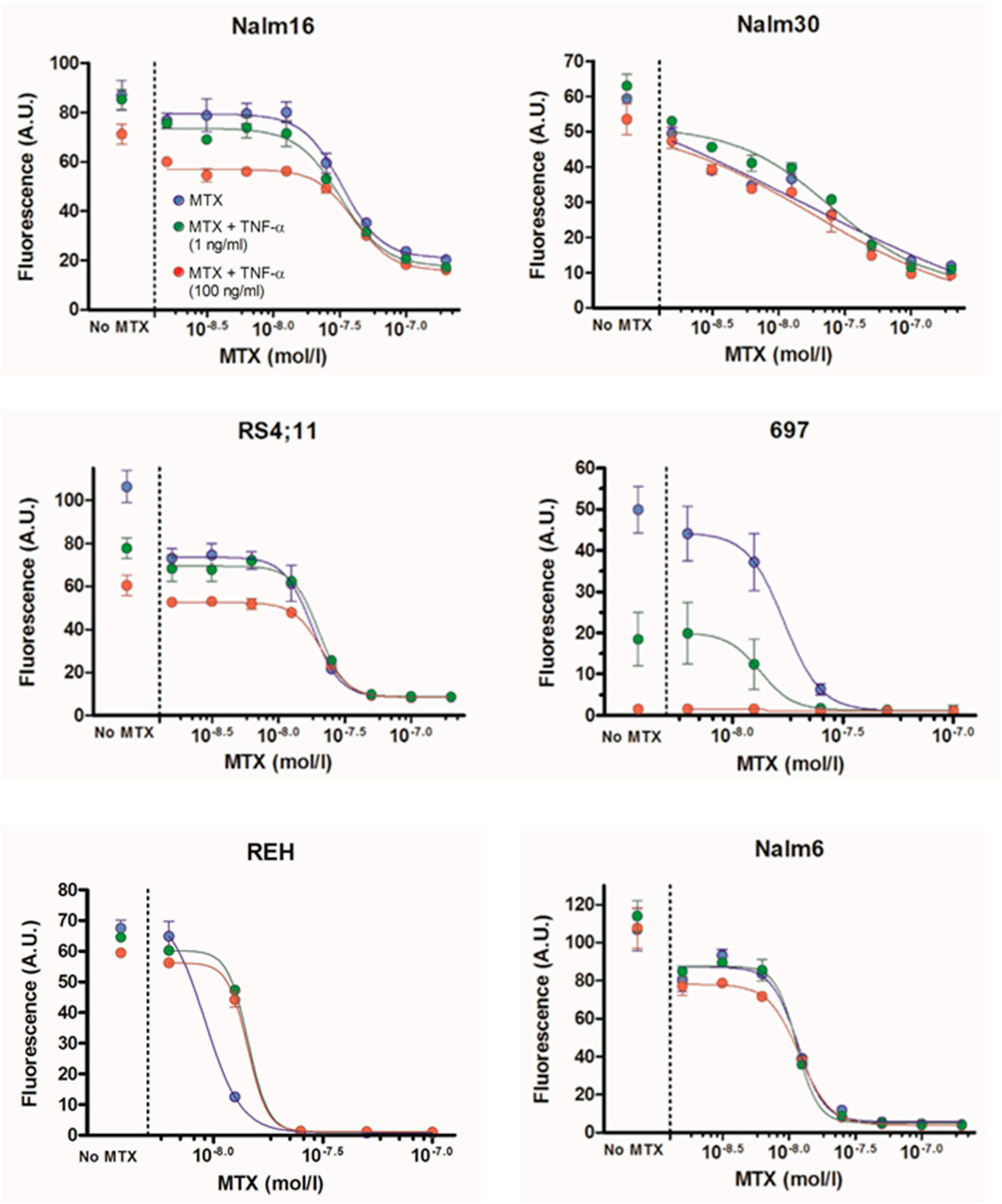

Figure S4. Survival curves to MTX in the presence or not of TNF-α. The cytokine was added daily over the course of 96 h (green: 1 ng/mL; red: 100 ng/mL; blue: only media). Abbreviations: ALL, acute lymphoblastic leukemia; A.U., arbitrary units; BCP-ALL, B-cell precursor acute lymphoblastic leukemia; MTX, methotrexate; T-ALL, T-cell acute lymphoblastic leukemia; TNF-α, tumor necrosis factor alpha.
